# Hemostatic Changes in Patients with COVID-19: A Meta-Analysis with Meta-Regressions

**DOI:** 10.3390/jcm9072244

**Published:** 2020-07-15

**Authors:** Matteo Nicola Dario Di Minno, Ilenia Calcaterra, Roberta Lupoli, Antonio Storino, Giorgio Alfredo Spedicato, Mauro Maniscalco, Alessandro Di Minno, Pasquale Ambrosino

**Affiliations:** 1 Department of Translational Medical Sciences, Federico II University, 80131 Naples, Italy; dario.diminno@hotmail.it; 2 Department of Clinical Medicine and Surgery, Federico II University, 80131 Naples, Italy; ileniacalcaterra@hotmail.it; 3 Department of Molecular Medicine and Medical Biotechnologies, Federico II University, 80131 Naples, Italy; roby.lupoli@gmail.com; 4 Istituti Clinici Scientifici Maugeri IRCCS, 27100 Pavia, Italy; antonio.storino@icsmaugeri.it (A.S.); mauro.maniscalco@icsmaugeri.it (M.M.); pasquale.ambrosino@icsmaugeri.it (P.A.); 5 Unipol Group, 40128 Bologna, Italy; spedicato_giorgio@yahoo.it; 6 Department of Pharmacy, Federico II University, 80131 Naples, Italy

**Keywords:** SARS-CoV-2, COVID-19, hemostasis, thrombosis, coagulation, disability, outcome

## Abstract

Background: Complications of coronavirus disease 2019 (COVID-19) include coagulopathy. We performed a meta-analysis on the association of COVID-19 severity with changes in hemostatic parameters. Methods: Data on prothrombin time (PT), activated partial thromboplastin time (aPTT), D-Dimer, platelets (PLT), or fibrinogen in severe versus mild COVID-19 patients, and/or in non-survivors to COVID-19 versus survivors were systematically searched. The standardized mean difference (SMD) was calculated. Results: Sixty studies comparing 5487 subjects with severe and 9670 subjects with mild COVID-19 documented higher PT (SMD: 0.41; 95%CI: 0.21, 0.60), D-Dimer (SMD: 0.67; 95%CI: 0.52, 0.82), and fibrinogen values (SMD: 1.84; 95%CI: 1.21, 2.47), with lower PLT count (SMD: −0.74; 95%CI: −1.01, −0.47) among severe patients. Twenty-five studies on 1511 COVID-19 non-survivors and 6287 survivors showed higher PT (SMD: 0.67; 95%CI: 0.39, 0.96) and D-Dimer values (SMD: 3.88; 95%CI: 2.70, 5.07), with lower PLT count (SMD: −0.60, 95%CI: −0.82, −0.38) among non-survivors. Regression models showed that C-reactive protein values were directly correlated with the difference in PT and fibrinogen. Conclusions: Significant hemostatic changes are associated with COVID-19 severity. Considering the risk of fatal complications with residual chronic disability and poor long-term outcomes, further studies should investigate the prognostic role of hemostatic parameters in COVID-19 patients.

## 1. Introduction

In December 2019, a cluster of patients with pneumonia of unknown origin was linked to a seafood wholesale market in Wuhan, China. A novel coronavirus, named 2019-nCoV or SARS-CoV-2, was isolated from human airway epithelial cells belonging to the subgenus sarbecovirus, Orthocoronavirinae subfamily [1]. Despite extensive control efforts implemented as part of a global containment strategy to minimize exportation, SARS-CoV-2 showed an international spread that led to a pandemic diffusion [2]. Epidemiologic data indicate that SARS-CoV-2 causes the Coronavirus Disease 2019 (COVID-19), a syndrome with a wide spectrum of clinical presentations [3,4,5]. In particular, the disease is characterized by fever, dyspnea, dry cough, and fatigue. Pulmonary imaging has shown multiple ground glass shadows and infiltrative shadows in both lungs. Severe cases have shown to develop into acute respiratory distress syndrome (ARDS) and septic shock [2,3,5,6], requiring specialized management at Intensive Care Units (ICU) [1,3,7,8,9,10], with poor long-term outcomes and residual chronic disability [11,12,13]. Coagulopathy is very common in patients in ICU and often indicates organ dysfunction or underlying diseases. In particular, disseminated intravascular coagulation (DIC) accompanies the clinical progression from systemic inflammatory response syndrome to severe sepsis and septic shock. In turn, progression to DIC leads to organ dysfunction, which is associated with increased mortality [14]. In patients without evidence of coagulopathy, higher D-dimer levels are associated with clinical severity among patients admitted to the ICU [15].

The aim of the present study is to perform a systematic review and meta-analyses to evaluate the association of COVID-19 severity with changes in hemostasis parameters. Moreover, we implemented some meta-regression models to evaluate the impact of demographic, clinical variables, and inflammatory markers on the evaluated outcomes.

## 2. Materials and Methods

We developed a protocol for this systematic review of literature data, defining the search strategy, the outcomes, the inclusion and exclusion criteria, and the statistical methods.

### 2.1. Data Sources and Searches

To detect all available studies on the association between hemostatic parameters and the severity of COVID-19, we conducted a systematic literature search in the main electronic databases (PubMed, Scopus, Web of Science, EMBASE) according to Preferred Reporting Items for Systematic Reviews and Meta-Analyses (PRISMA) guidelines [16]. The last search was performed on 16 June 2020 with no language restriction, by using the following terms in any possible association; COVID-19, SARS-CoV-2, coagulative, coagulation, hemostatic, hemostasis, prothrombin time, activated partial thromboplastin time, D-Dimer, platelet, and fibrinogen.

Moreover, the reference lists of all included articles were manually consulted. In case of missing data among studies fulfilling the inclusion criteria, the authors were contacted by e-mail to try to claim the original data. Two authors (RL and AS) analyzed each article and separately performed the extraction of data. In case of disagreement, a third investigator was consulted (ADM). Discrepancies were resolved by consensus. Overall, selection results showed a high inter-reader agreement (κ = 1.00) and were reported according to PRISMA flowchart (Supplemental Figure S1).

### 2.2. Study Selection

According to the aforementioned protocol, all studies reporting data about the association of hemostatic parameters with COVID-19 severity were included. Case reports, reviews, and articles on animal models were excluded.

### 2.3. Data Extraction and Quality Assessment

We included in the analysis studies providing data on prothrombin time (PT), activated partial thromboplastin time (aPTT), D-Dimer, platelet count (PLT), or fibrinogen; in study group 1: severe COVID-19 (cases) and mild COVID-19 patients (controls) and in study group 2: subjects dead with COVID-19 (cases) and subjects survived to COVID-19 (controls). 

In each study, besides hemostatic parameters, data regarding sample size, mean age of enrolled subjects, prevalence of male sex, mean C-reactive protein (CRP) levels, prevalence of diabetes, and prevalence of hypertension were extracted. Formal quality score adjudication was not used as most included studies were case series or small cohort studies.

### 2.4. Data Synthesis and Analysis

Data synthesis and analyses were performed by using comprehensive meta-analysis (Version 2, Biostat, Englewood, NJ, USA, 2005). Because of the heterogeneity in laboratory techniques, reference values, and units of measurement, differences in PT, aPTT, D-Dimer, PLT, and fibrinogen between cases and controls were expressed as standardized mean difference (SMD) with 95% confidence intervals (95%CI), which represents the difference between the weighted mean and the standard deviation of the outcome in cases and controls.

For all evaluated outcomes, data from study group 1 and study group 2 were separately analyzed. The pooled effect was tested using Z-scores, with *p* < 0.05 being considered statically significant. We evaluated statistical heterogeneity among studies with chi-squared Cochran’s Q test and with I^2^ index, which measures the inconsistency among results of studies and defines the proportion of total variation in study estimates that is due to heterogeneity rather than sampling error. In particular, an I^2^ value of 25% corresponds to low, 25–50% to moderate, and 50% to high heterogeneity [17]. Funnel plots of the standard difference in means vs. the standard error were used as a graphical representation of publication bias. To detect a potential small-study effect, visual inspection of funnel plots asymmetry was performed. Moreover, the Egger’s test was used to assess publication bias over and above any subjective evaluation, with a *p* < 0.10 being considered statistically significant [18]. In case of a significant publication bias, the Duval and Tweedie’s trim and fill method allowed for the assessment of the adjusted effect size [19]. 

To be as conservative as possible, the random-effect method was used to take into account the heterogeneity among the included studies.

To evaluate whether demographic variables (mean age and prevalence of male sex) and clinical data (CRP levels, prevalence of diabetes, and prevalence of hypertension) may impact on differences in the hemostatic outcomes between cases and controls, we implemented multiple meta-regression analyses with differences in PT, aPTT, D-Dimer, PLT, and fibrinogen as dependent variables (y) and the above-mentioned covariates as independent variables (x). Comprehensive meta-analysis (Version 2, Biostat, Englewood, NJ, USA, 2005) was used for the multivariate approach.

## 3. Results

After excluding duplicate results, the search retrieved 518 articles. Of them, we excluded 194 because they were found to be off topic after scanning the title and/or the abstract, and 197 reviews/comments/case reports or studies lacking data of interest. Another 43 studies were eliminated after a full evaluation of the texts. Four studies [20,21,22,23] reported data on the same study sample. Thus, most recent studies with the largest samples were included in the analysis [20,23] (Supplemental Figure S1).

Overall, 84 studies were included in the final analyses. Of them, 60 studies reported on a total of 5487 subjects with severe COVID-19 and 9670 subjects with mild COVID-19 [6,8,24,25,26,27,28,29,30,31,32,33,34,35,36,37,38,39,40,41,42,43,44,45,46,47,48,49,50,51,52,53,54,55,56,57,58,59,60,61,62,63,64,65,66,67,68,69,70,71,72,73,74,75,76,77,78,79,80,81].

In addition, 25 studies on a total of 1511 subjects dead with COVID-19 and 6287 subjects survived to COVID-19 were included in the meta-analysis [20,23,26,74,82,83,84,85,86,87,88,89,90,91,92,93,94,95,96,97,98,99,100,101,102].

### 3.1. Study Characteristics

Principal characteristics of subjects enrolled in included studies are shown in Table 1. The number of patients varied from 17 to 4468, the male sex represented 53.9% of the study population (range: 9.1–81.0%), and the mean age was 54.4 years (range: 35.6–70.7). Diabetes was found in 16.5% (range: 3.2–100%) of patients and hypertension in 28.7% (range: 5.0–83.0%). The mean value of CRP in patients enrolled was 47.7 mg/L (range: 3.14–192.0).

### 3.2. Study Group 1 (Severe COVID-19 vs. Mild COVID-19)

**Figure 1:**
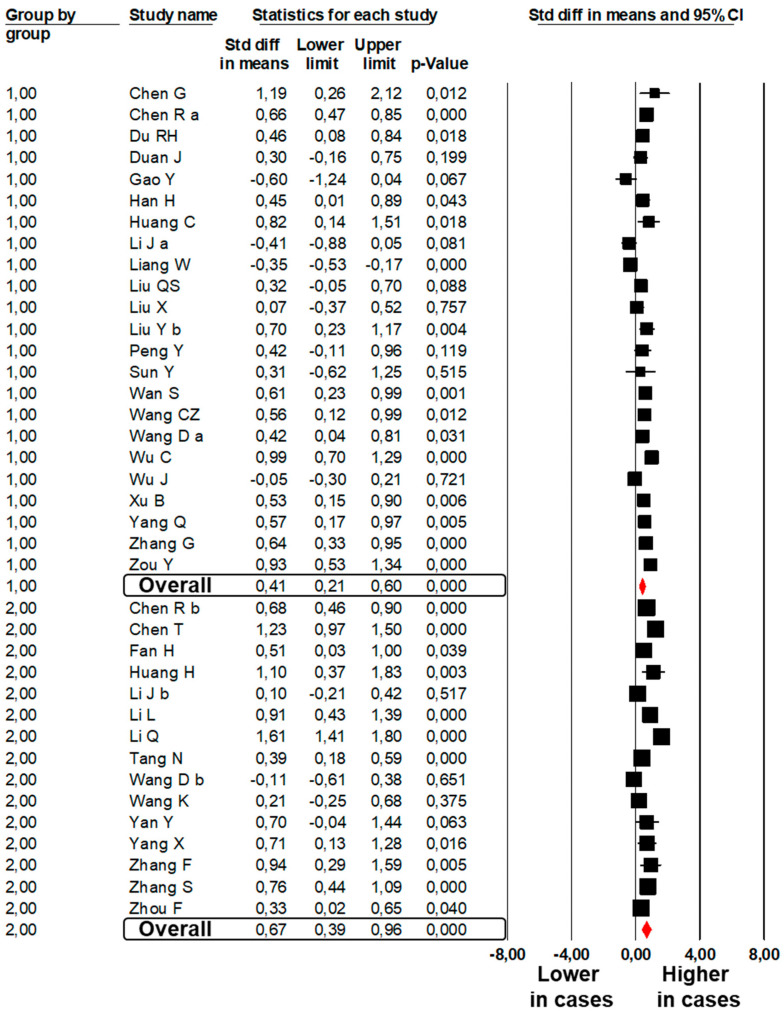
Forest plots of the difference in prothrombin time values between subjects with severe and those with mild COVID-19 (Group 1) and between non-survivors and survivors to COVID-19 (Group 2).

A total of 23 studies [6,8,25,26,31,32,35,38,40,41,44,45,46,54,61,63,64,67,68,70,73,76,81] showed significantly higher PT values in 1041 subjects with severe COVID-19 as compared to 3835 subjects with mild COVID-19 (SMD: 0.41; 95%CI: 0.21, 0.60, *p* < 0.001, Figure 1). The heterogeneity among studies was significant (I^2^: 84.3%, *p* < 0.001) and was not reduced by the exclusion of one study at a time. 

**Figure 2:**
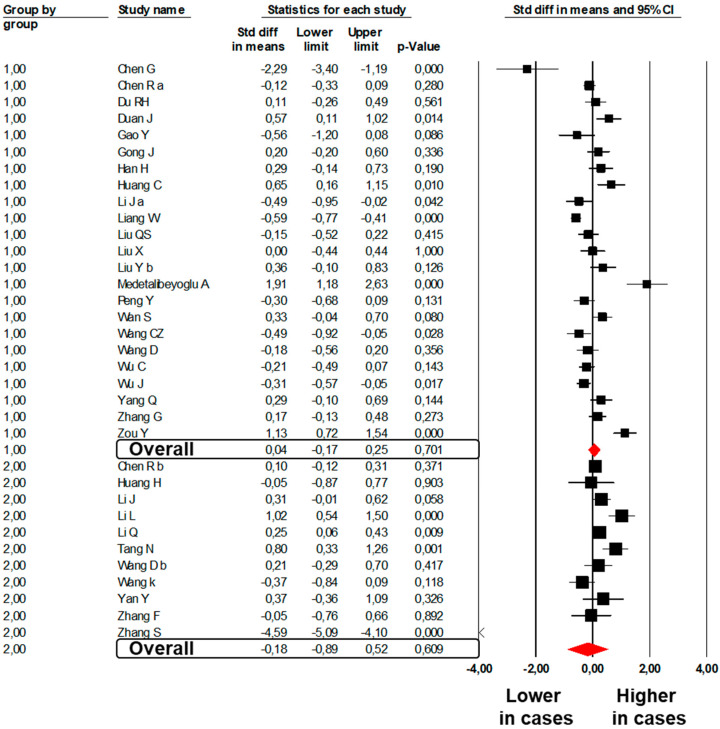
Forest plots of the difference in activated partial thromboplastin time values between subjects with severe and those with mild COVID-19 (Group 1) and between non-survivors and survivors to COVID-19 (Group 2).

In contrast, no difference was found in 23 studies [6,8,25,26,31,32,35,36,38,40,41,44,45,46,51,54,63,64,67,68,73,76,81] reporting aPTT values between 1018 subjects with severe COVID-19 and 3976 subjects with mild disease (SMD: 0.04; 95%CI: −0.17, 0.25, *p* = 0.701; I^2^: 86.1%, *p* < 0.001, Figure 2).

**Figure 3:**
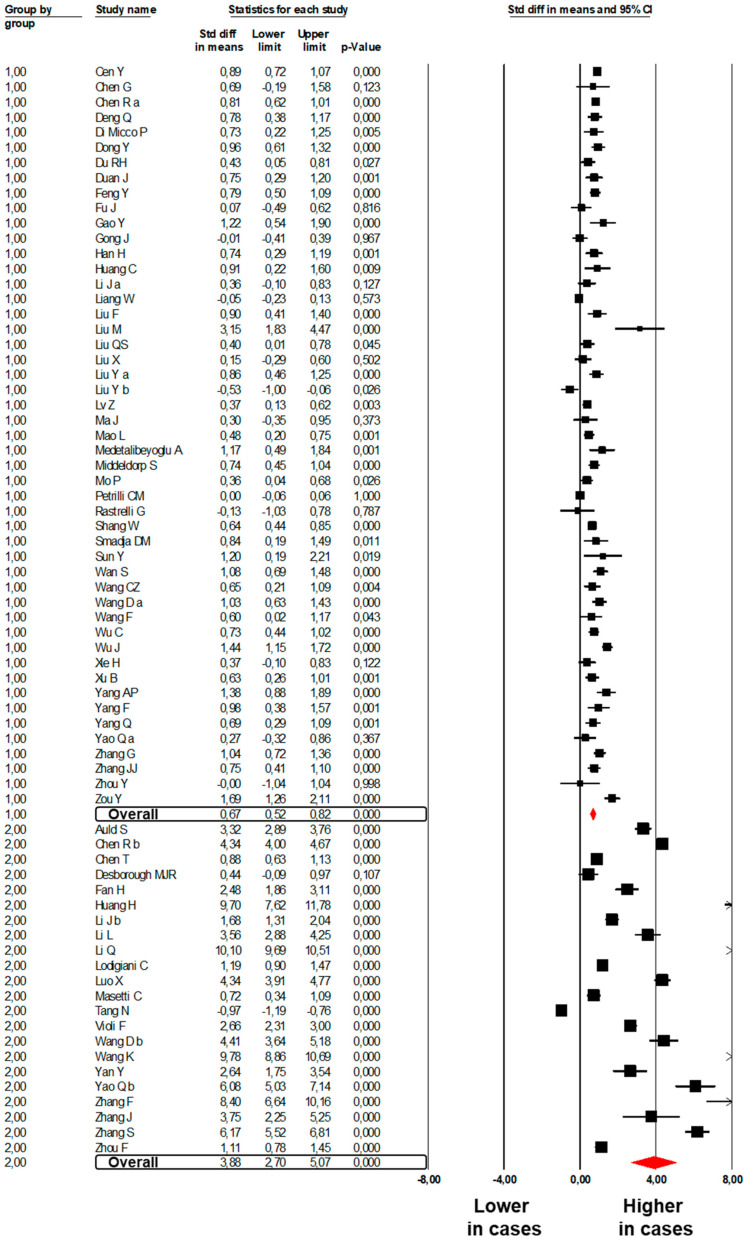
Forest plots of the difference in D-Dimer values between subjects with severe and those with mild COVID-19 (Group 1) and between non-survivors and survivors to COVID-19 (Group 2).

Forty-nine studies [6,8,24,25,26,27,28,30,31,32,33,34,35,36,38,41,42,43,44,45,46,47,48,49,50,51,52,53,55,57,58,59,61,63,64,65,67,68,69,70,71,72,73,74,76,77,80,81,87] showed higher D-Dimer levels in 5024 subjects with severe COVID-19 than in 8052 subjects with mild COVID-19 (SMD: 0.67; 95%CI: 0.52, 0.82, *p* < 0.001, Figure 3). Heterogeneity was significant (I^2^: 90.4%, *p* < 0.001) and was not reduced by the exclusion of one study at a time.

**Figure 4:**
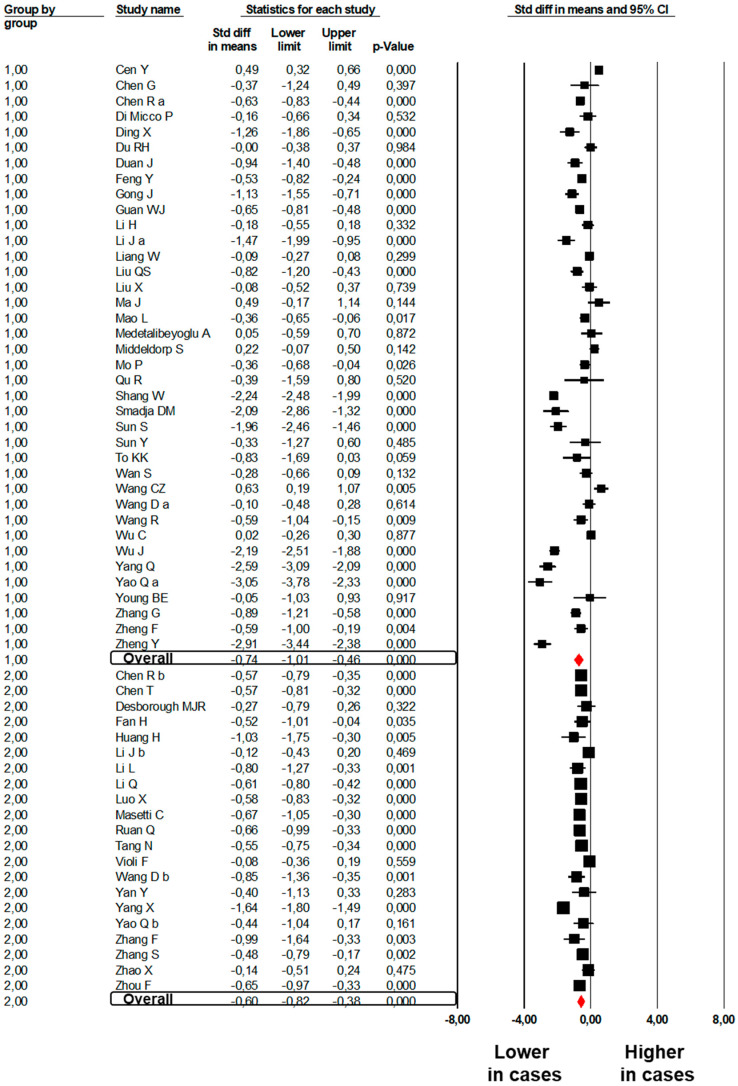
Forest plots of the difference in platelet count between subjects with severe and those with mild COVID-19 (Group 1) and between non-survivors and survivors to COVID-19 (Group 2).

Significantly lower PLT count (SMD: −0.74; 95%CI: −1.01, −0.47, *p* < 0.001, Figure 4) was found between 1956 subjects with severe COVID-19 as compared to 6546 mild subjects enrolled in 38 studies [6,24,25,26,28,29,31,32,33,36,37,39,41,44,45,49,50,51,52,53,56,58,59,60,61,62,63,64,66,67,68,73,74,75,76,78,79,87]. The heterogeneity among studies was significant (I^2^: 95.5%, *p* < 0.001) and was not reduced after excluding one study at a time.

**Figure 5:**
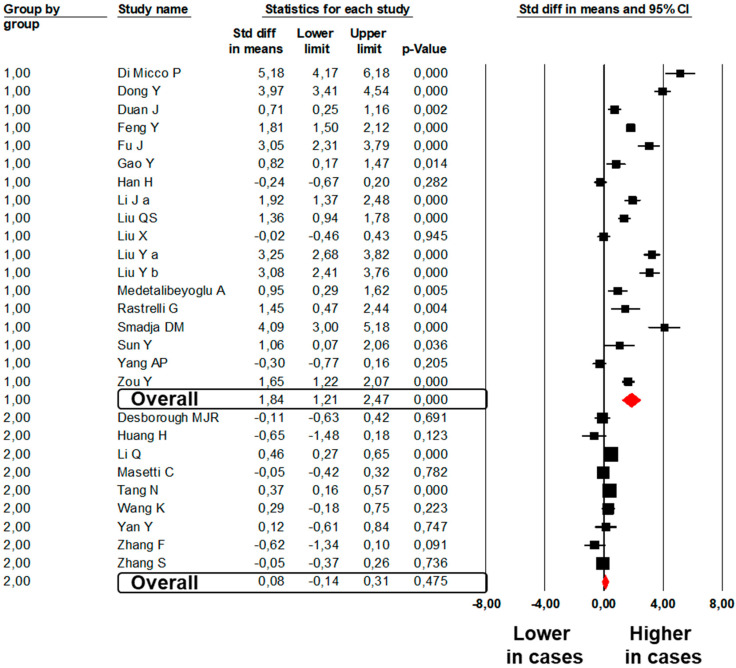
Forest plots of the difference in fibrinogen values between subjects with severe and those with mild COVID-19 (Group 1) and between non-survivors and survivors to COVID-19 (Group 2).

A total of 18 studies [28,30,32,33,34,35,38,40,44,45,46,47,51,57,59,61,71,81] showed significantly higher fibrinogen values in 469 subjects with severe as compared to 1434 subjects with mild COVID-19 (SMD: 1.84; 95%CI: 1.21, 2.47, *p* < 0.001, Figure 5). The heterogeneity among studies was significant (I^2^: 95.8%, *p* < 0.001) and was not reduced by the exclusion of one study at a time.

### 3.3. Study Group 2 (Dead with COVID-19 vs. Survived to COVID-19) 

A total of 15 studies [20,23,26,83,85,86,87,88,89,95,96,97,98,100,102] showed significantly higher PT values in 840 subjects dead with COVID-19 as compared to 3287 subjects survived to COVID-19 (SMD: 0.67; 95%CI: 0.39, 0.96, *p* < 0.001, Figure 1). The heterogeneity among studies was significant (I^2^: 90.2%, *p* < 0.001) and was not reduced by the exclusion of one study at a time. 

No significant difference was found in 11 studies [20,26,86,87,88,89,95,96,97,98,100] reporting aPTT values between 475 subjects dead with COVID-19 and 2774 subjects survived to COVID-19 (SMD: −0.18; 95%CI: −0.89, 0.52 *p* = 0.609; I^2^: 97.3%, *p* < 0.001, Figure 2).

Twenty-two studies [20,26,74,82,83,84,85,86,87,88,89,90,91,92,94,95,96,97,98,99,100,102] showed higher D-Dimer levels in 1149 subjects dead with COVID-19 as compared to 4407 subjects survived to COVID-19 (SMD: 3.88; 95%CI: 2.70, 5.07, *p* < 0.001, Figure 3). The heterogeneity among studies was significant (I^2^: 99.4%, *p* < 0.001) and was not reduced by the exclusion of one study at a time. 

A lower PLT count (SMD: −0.60, 95%CI: −0.82, −0.38; *p* < 0.001, Figure 4) was found in 1339 non-survivors to COVID-19 as compared to 5637 survivors enrolled in 21 studies [20,22,26,74,83,84,85,86,87,88,89,91,92,93,94,95,97,98,100,101,102]. The heterogeneity among studies was significant (I^2^: 90.3%, *p* < 0.001) and was not reduced by the exclusion of one study at a time. 

A total of nine studies [20,84,86,89,92,96,97,98,100] showed comparable fibrinogen values in 429 subjects dead with COVID-19 as compared to 2472 subjects survived (SMD: 0.08; 95%CI: −0.14, 0.31, *p* = 0.475, Figure 5). The heterogeneity among studies was significant (I^2^: 67.1%, *p* = 0.002) and was not reduced by the exclusion of one study at a time.

### 3.4. Publication Bias

Visual inspection of funnel plots and the Egger’s test suggested the absence of publication bias and of small-study effect for studies evaluating PT, aPTT, and PLT. In contrast, a significant publication bias was found for D-Dimer and fibrinogen, confirmed by the Egger’s test (Egger’s *p* < 0.001 and *p* = 0.039, respectively). Of interest, the Duval and Tweedie’s trim and fill analysis substantially confirmed all results for D-Dimer (SMD: 2.08; 95%CI: 1.63, 2.53) and fibrinogen (SMD: 1.34; 95%CI: 0.91, 1.78) after trimming and imputing studies (Supplemental Figure S2).

### 3.5. Meta-Regression Analyses

Meta-regression models showed an impact of CRP levels on the difference in PT (Z-value: 2.27, *p* = 0.023) and fibrinogen (Z-value: 2.89, *p* = 0.004). Male sex was associated with a lower difference in D-Dimer (Z-value: −2.43, *p* = 0.015) and PLT levels (Z-value: 1.97, *p* = 0.048). A higher difference in fibrinogen was observed for increasing prevalence of hypertension (Z-value: 2.63, *p* = 0.009) (Supplemental Table S1).

## 4. Discussion

Results of the present meta-analysis consistently show that patients with severe COVID-19 exhibit higher PT, D-Dimer, and fibrinogen values, with a lower PLT count than subjects with mild disease. A significant difference for PT, D-Dimer, and PLT was also found in non-survivors to COVID-19, with similar fibrinogen levels as compared to survivors. Regression models further refined results, showing that differences in PT and fibrinogen levels directly correlated with the degree of inflammation, as expressed by CRP values. Moreover, prevalence of male sex significantly impacted the difference in D-Dimer levels and PLT count. Last, a higher difference in fibrinogen was observed for increasing prevalence of hypertension.

In the attempt to identify new biomarkers of disease severity, some meta-analytical studies have been performed so far on a similar topic, with contrasting results. Our findings are partially consistent with those of a previous meta-analysis [103], documenting significantly higher PT and D-Dimer levels in patients with severe COVID-19. However, differing from our findings, the authors documented no significant difference in PLT levels between severe and mild patients. Two further meta-analyses were specifically focused on PLT, showing that a low PLT count is associated with an increased risk of severe disease and mortality in patients with COVID-19 [104,105]. Overall, literature data about hemostatic parameters and COVID-19 have rapidly grown and these previous meta-analyses included a limited number of available studies. Here, we report the largest meta-analysis (84 studies) including multiple hemostatic parameters, with the aim to provide a comprehensive overview on the association between COVID-19 severity and hemostatic changes.

In the past decade, thrombocytopenia was documented in more than 50% of patients infected with SARS, being this laboratory finding identified as a significant predictor of mortality [106,107] with a high prognostic value [108]. Thrombocytopenia is common in several critical conditions, suggesting the presence of severe organ dysfunction and, in some cases, the development of intravascular coagulopathy [109]. Besides thrombocytopenia, our study suggests that both severe patients and non-survivors to COVID-19 exhibit significantly higher values of D-Dimer and prolonged PT. 

The prognostic role of PT has been evaluated in several clinical settings, being identified as a predictor of death after myocardial infarction [110,111], after surgical intervention [112], in acute decompensated heart failure [113] or in neoplastic diseases [114]. PT is commonly used to evaluate the extrinsic and common pathways of coagulation, thus detecting low fibrinogen concentrations and deficiencies of factors II, V, VII, and X [115]. On the other hand, aPTT evaluates the function of all clotting factors with the only exception of factor VII [115]. As factor VII is the one with the shortest half-life, PT is the first hemostatic parameter affected in many clinical conditions [116]. 

In our meta-analysis, we documented a significant prolongation of PT, without significant modifications in aPTT. In this regard, some hypotheses can be done, such as the highly increased factor VIII activity [117] correcting an otherwise prolonged aPTT [118]. However, considering the hepatic origin of clotting factors and the short half-life of factor VII [119], we can also hypothesize that a prolonged PT with normal aPTT may be due to acute liver dysfunction. This is in line with the observed association between altered liver markers and COVID-19 severity [120]. Recent studies demonstrated that patients with severe COVID-19 have increased incidence of abnormal liver function, with significantly higher values of transaminases as compared to subjects with a mild disease [25,37]. In keeping with this, a recent meta-analysis confirmed the presence of hepatic impairments in SARS and MERS Coronaviridae infections [121]. Several hypotheses have been made to explain the presence of liver injury in severe COVID-19 patients, such as the fact that a high positive end-expiratory pressure may determine hepatic congestion through an increase in right atrial pressure [120]. However, the hypothesis of a concomitant liver damage from virally-induced cytotoxic T cells seems to be the most convincing [120]. 

The observation of higher PT values both in severe COVID-19 patients and non-survivors could also be interpreted from an alternative (or additional) point of view. In 2003, a total of 2.5% of SARS patients showed evidence of disseminated intravascular coagulation (DIC), being this condition frequently associated with death [122]. Accordingly, among 183 in-hospital patients with COVID-19, 71.4% of non-survivors had overt DIC as compared to only 0.6% of survivors [21]. The development of a consumption coagulopathy in severe COVID-19 may account also for the lower PLT count and the increased D-Dimer levels documented in our meta-analysis, in line with the laboratory criteria for overt DIC proposed by the International Society of Thrombosis and Hemostasis (ISTH) [123]. However, most patients with severe COVID-19 may not fulfill ISTH criteria for DIC, as coagulopathy seems to have its specific features in this clinical setting. For example, thrombocytopenia is less pronounced in severe COVID-19 patients as compared to sepsis-related DIC, while D-Dimer levels are exceptionally high [124]. Moreover, we documented higher fibrinogen levels in severe patients when compared with mild subjects, thus refuting the hypothesis of a consumption coagulopathy in this clinical setting. In line with previous evidence [117], this finding is further supported by the lack of changes in aPTT values both in severe and in COVID-19 non-survivors.

A further interesting finding is the direct correlation between CRP and PT values. CRP levels correlate with the intensity of the systemic inflammatory response and, since hemostasis and inflammation are strictly related [125], this supports the hypothesis of a systemic activation of the coagulation system in response to a dysregulation of the inflammatory markers. This is also in agreement with the increase in fibrinogen levels, mainly documented in most severe patients.

Taken together, the currently available evidence seems to suggest that SARS-CoV-2 infection can rapidly evolve into a severe condition with pulmonary and multiorgan complications, potentially resulting in a consistent hemostatic imbalance with hypercoagulable state. However, the exact mechanisms behind the observed hemostatic changes in severe COVID-19 patients are still unclear. It has been hypothesized that the coagulopathy in SARS-CoV-2 infection may be a combination of a low-grade DIC with its distinct features and a localized pulmonary thrombotic microangiopathy [124]. Alternatively, the hemostatic imbalance has been associated to the severe inflammatory state [117]. The high frequency of antiphospholipid antibodies documented among critically ill COVID-19 patients has also been related to the genesis of the hypercoagulable state [126,127]. In the meantime, a growing amount of literature data suggests that a high incidence of thromboembolic complications is expected among severe and critical patients with COVID-19, even in the presence of adequate thromboprophylaxis [128,129]. 

Further cohort studies are needed to clarify the pathogenesis and the prognostic value of the hemostatic changes associated to SARS-CoV-2 infection. This could allow for the identification of new markers of disease progression. Moreover, considering the unknown long-term outcomes of COVID-19, the growing amount of studies suggesting the presence of a residual chronic disability, and the need of post-discharge rehabilitation after the acute phase [130,131,132,133,134,135], it is essential to identify new biomarkers related to the disease for an adequate management of critical patients even after ICU (e.g., general ward, rehabilitation centers, and home). The hypothesis that persistent thromboembolic phenomena may contribute to the long-term outcomes of COVID-19 [136,137] and to the functional sequelae of critically ill patients after discharge [12,129] further supports the urgent need of studies addressing the mechanisms of such hemostatic imbalance in this clinical setting.

Some potential limitations of our study need to be discussed. First, studies included in our meta-analysis enrolled patients with different characteristics, since these studies had different inclusion and exclusion criteria. Moreover, considerable information is missing in included studies. Considering that meta-analyses are performed on pooled data, our meta-regression models allowed for the adjustment for some—but not all—potential confounders. Thus, although the multivariate approach was able to refine analyses by assessing the influence of most clinical and demographic variables on the observed results, our findings should be interpreted with great caution. 

Second, most results of our meta-analysis were affected by significant heterogeneity. Although it was not possible to definitively identify the origin of such heterogeneity, the impact of major clinical and demographic variables on our results was evaluated through regression models. Moreover, we excluded the presence of publication bias by using different methods.

Finally, we performed our analyses by using SMD instead of mean difference, this method being designed to be used when the same outcome is evaluated in a variety of ways [138]. This allowed us to provide consistent meta-analytical information with an appropriate statistical methodology, but we were not able to provide a quantitative measure of the difference in the hemostatic parameters between cases and controls. This partially reduces the clinical relevance of our results.

In conclusion, our meta-analysis suggests that significant hemostatic changes are associated with COVID-19 severity. Strict monitoring of these parameters is needed to identify early modifications with the aim to stratify the death risk and to implement adequate therapeutic approaches. Considering the risk of fatal complications and poor long-term outcomes, further studies should better define the prognostic role of hemostatic parameters in COVID-19 patients.

## Figures and Tables

**Table 1:** Clinical and demographic characteristics of patients with COVID-19 included in different studies.

**Study Group 1—Mild Disease vs. Severe Disease**											
**Author**	**N of Patients (*n*)**	**Male Gender (%)**	**Age (Years)**	**CRP (mg/L)**	**Diabetes (%)**	**Hypertension (%)**	**Reported Outcome**				
							**PT**	**aPTT**	**D-Dimer**	**PLT**	**FYB**
Cen Y	652	--	--	17.53	--	--	NO	NO	YES	YES	NO
Chen G	21	81	57	92	14.3	23.8	YES	YES	YES	YES	NO
Chen R a	500	57.1	56	38.30	11.1	27	YES	YES	YES	YES	NO
Deng Q	112	50.9	62.45	88.00	17	32.1	NO	NO	YES	NO	NO
Di Micco P	67	70		14.60			NO	NO	YES	YES	YES
Ding X	72	45.8	49.75		6.9	12.5	NO	NO	NO	YES	NO
Dong Y	147	42.9	47.00	22.62			NO	NO	YES	NO	YES
Du RH	109	67.9	70.7	85.70	31.2	59.6	YES	YES	YES	YES	NO
Duan J	348	52.9	45	11.29	3.2	7.8	YES	YES	YES	YES	YES
Feng Y	406	56.9	52.50	24.96	10.3	23.7	NO	NO	YES	YES	YES
Fu J	75	60	46.6	59.56	5.3	9.3	NO	NO	YES	NO	YES
Gao Y	43	60.5	44.4	32.2	--	--	YES	YES	YES	NO	YES
Gong J	189	46.6	48.79	--	--	--	NO	YES	YES	YES	NO
Guan W	1099	9.1	46.4	--	7.4	15	NO	NO	NO	YES	NO
Han H	84	--	--	--	--	--	YES	YES	YES	NO	YES
Huang C	41	73.2	49.5	--	19.5	14.6	YES	YES	YES	NO	NO
Li H	116	56.8	62.05	54.93	--	--	NO	NO	NO	YES	NO
Li J a	75	55.97	59.50	3.14	--	32.84	YES	YES	YES	YES	YES
Liang W	1590	57.3	48.9	34.80	8.2	16.9	YES	YES	YES	YES	NO
Liu F	134	47	51.5	--	7.5	20.1	NO	NO	YES	NO	NO
Liu M	30	--	--	--	--	--	NO	NO	YES	NO	NO
Liu QS	150	52.7	42.19		11.3	19.3	YES	YES	YES	YES	YES
Liu X	112	--	56	46	--	--	YES	YES	YES	YES	YES
Liu Y a	109	54.1	54.5	33.3	11	33.9	NO	NO	YES	NO	YES
Liu Y b	76	64.5	46.50	--	--	--	YES	YES	YES	NO	YES
Lv Z	270	49.44	59.25	47.23	9.89	20.9	NO	NO	YES	NO	NO
Ma J	37	54.1	63.25	--	--	--	NO	NO	YES	YES	NO
Mao L	214	40.7	52.7	--	--	--	NO	NO	YES	YES	NO
Medetalibeyoğlu A	68	69.1	56.52	--	--	--	NO	YES	YES	YES	YES
Middeldorp S	198	65.7	60.8	--	--	--	NO	NO	YES	YES	NO
Mo P	155	55.5	53.9	43.9	9.7	23.9	NO	NO	YES	YES	NO
Peng Y	112	47.3	58.6	49.9	--	83	YES	YES	NO	NO	NO
Petrilli CM	4468	60.2	--	102.84	33.8	60.6	NO	NO	YES	NO	NO
Qu R	30	--	58.9	--	--	--	NO	NO	NO	YES	NO
Rastrelli G	27	--	62.60	26.63	29.6	51.8	NO	NO	YES	NO	YES
Shang W	443	49.7	55.50	23.77	14.2	29.6	NO	NO	YES	YES	NO
Smadja DM	40		56.52	110.60	20	40	NO	NO	YES	YES	YES
Sun S	116	51.7	49.50	--	--	--	NO	NO	NO	YES	NO
Sun Y	18	--	--	--	--	--	YES	NO	YES	YES	YES
To KK	23	56.5	59.00	--	17	26	NO	NO	NO	YES	NO
Wan S	135	54.1	47.4	37.2	8.9	9.6	YES	YES	YES	YES	NO
Wang CZ	85	52.9	59.40	43.27	11.8	25.9	YES	YES	YES	YES	NO
Wang D a	138	45.7	54.4	--	10.1	31.2	YES	YES	YES	YES	NO
Wang F	50	57	57.11	68.88	--	--	NO	NO	YES	NO	NO
Wang R	125	56.8	38.76	17.76	--	--	NO	NO	NO	YES	NO
Wu C	201	63.7	52.3	55.3	10.9	19.4	YES	YES	YES	YES	NO
Wu J	280	53.93	43.12	7.33	--	--	YES	YES	YES	YES	NO
Xie H	79	55.7	58.50	20.70	10.1	17.7	NO	NO	YES	NO	NO
Xu B	125	55	60.88	32.69	--	26.7	YES	NO	YES	NO	NO
Yang AP	93	62.2	46.4	--	22.5	24.7	NO	NO	YES	NO	YES
Yang F	52	53.8	64.50	30.80	--	--	NO	NO	YES	NO	NO
Yang Q	136	48.5	55.00	42.03	14.7	27.1	YES	YES	YES	YES	NO
Yao Q a	108	39.8	49.75	13.13	4.6	14.8	NO	NO	YES	YES	NO
Young BE	18	50	47.1	30.3	5.6	22.2	NO	NO	NO	YES	NO
Zhang G	221	48.9	53.88	--	24.4	35.8	YES	YES	YES	YES	NO
Zhang JJ	140	50.7	57.0	34.2	12.1	30	NO	NO	YES	NO	NO
Zheng F	161	49.7	45.13	20.15	4.3	13.7	NO	NO	NO	YES	NO
Zheng Y	141	52.4	47.00	--	--	--	NO	NO	NO	YES	NO
Zhou Y	17	35.3	--	--	--	--	NO	NO	YES	NO	NO
Zou Y	303	52.1	51.2	--	--	--	YES	YES	YES	NO	YES
**Study Group 2—Deaths vs. Survivors**											
**Author**	**N of Patients (*n*)**	**Male Gender (%)**	**Age (Years)**	**CRP (mg/L)**	**Diabetes (%)**	**Hypertension (%)**	**Reported Outcome**				
							**PT**	**aPTT**	**D-Dimer**	**PLT**	**FYB**
Auld S	217	54.8	63.75	192.00	45.6	61.7	NO	NO	YES	NO	NO
Chen R b	548	57.1	56	38.3	11.1	27	YES	YES	YES	YES	NO
Chen T	274	62.4	58.6	61.7	17.2	33.9	YES	NO	YES	YES	NO
Desborough MJR	66	73	58.25	190.25	41	45	NO	NO	YES	YES	YES
Fan H	73	67.12	58.36	93.84	--	32.88	YES	NO	YES	YES	NO
Huang H	50	46	35.6	30.64	--	--	YES	YES	YES	YES	YES
Li J b	161	--	55.40	--	--	--	YES	YES	YES	YES	NO
Li L	93	44	51	14.80	--	5	YES	YES	YES	YES	NO
Li Q	1449	51	55.5	--	--	--	YES	YES	YES	YES	YES
Lodigiani C	285	--	--	--	--	--	NO	NO	YES	NO	NO
Luo X	298	50.3	55.75	34.03	15.1	28.9	NO	NO	YES	YES	NO
Masetti C	229	64.6	60.7	8.6	18.8	38	NO	NO	YES	YES	YES
Ruan Q	150	68.0	56.5	76	16.7	34.7	NO	NO	NO	YES	NO
Tang N	449	59.7	65.1	--	--	--	YES	YES *	YES	YES	YES
Violi F	319	60.4	65.61	65.98	18.6	54.6	NO	NO	YES	YES	NO
Wang D b	107	53.3	50.75		10.3	24.3	YES	YES	YES	YES	NO
Wang K	296	47.3	47.32	15.05	10.1	14.2	YES	YES	YES	NO	YES
Yan Y	48	68.8	69.4	--	100	50	YES	YES	YES	YES	YES
Yang X	1476	52.6	57	--	--	--	YES *	NO	NO	YES	NO
Yao Q b	108	39.8	49.75	13.13	4.6	14.8	NO	NO	YES	YES	NO
Zhang F	53	25.8	--	29.14	--	--	YES	YES	YES	YES	YES
Zhang J	19	57.9	68.75	106.79	--	--	NO	NO	YES	NO	NO
Zhang S	315	55.55	56	39.13	13.02	24.76	YES	YES	YES	YES	YES
Zhao X	532	46.2	49.10	--	11.1	20.3	NO	NO	NO	YES	NO
Zhou F	191	36.1	56.7	--	18.8	30.4	YES	NO	YES	YES	NO

Legend: CRP: C-reactive protein; PT: prothrombin time; aPTT: activated partial thromboplastin time; PLT: platelet count. * Data derived by [21,22].

## Supplementary Materials

The following are available online at https://www.mdpi.com/2077-0383/9/7/2244/s1, Figure S1: Preferred Reporting Items for Systematic Reviews and Meta-Analyses (PRISMA) flow diagram, Figure S2: Funnel plots of effect size versus standard error for studies evaluating the difference in PT, aPTT, D-Dimer, platelet count and fibrinogen, Table S1: Meta-regression analyses. Impact of age, male gender, hypertension, diabetes, and C-reactive protein (CRP) on the difference in PT, D-Dimer, platelet count, and fibrinogen levels, Table S2: PubMed search (June 16, 2020).
