# Supplementary material for: Hemostatic Changes in Patients with COVID-19: A Meta-Analysis with Meta-Regressions
Source: J Clin Med. 2020 Jul 15;9(7):2244. doi: 10.3390/jcm9072244 (PMC7408674; doi:10.3390/jcm9072244)
Supplement: Supplementary file 1 [file jcm-09-02244-s001.pdf]

Journal of Clinical Medicine

Supplementary online material

Haemostatic changes in patients with COVID-19: a meta-analysis with meta-regressions.

Matteo Nicola Dario Di Minno, MD PhD[1] Ilenia Calcaterra, MD[2] Roberta Lupoli, MD PhD[3] Antonio Storino, MD[4] Giorgio Alfredo Spedicato, PhD FCAS FSA CSPA CStat[5] Mauro Maniscalco, MD PhD[4] Alessandro Di Minno, PharmD PhD[6]* Pasquale Ambrosino, MD PhD[4]*

1 Department of Translational Medical Sciences, Federico II University, Naples, Italy.
2 Department of Clinical Medicine and Surgery, Federico II University, Naples, Italy;
3 Department of Molecular Medicine and Medical Biotechnologies, Federico II University, Naples, Italy;
4 Istituti Clinici Scientifici Maugeri IRCCS, Pavia, Italy;
5 Unipol Group, Bologna, Italy;
6 Department of Pharmacy, Federico II University, Naples, Italy.
*** The two Authors share co-seniorship.**

Correspondence: Alessandro Di Minno, PharmD PhD; Department of Pharmacy, Federico II University, Naples, Italy; Tel/Fax: +39.081.7464323; alessandro.diminno@unina.it

Table of contents:

**Supplemental Figure 1**	**Preferred Reporting Items for Systematic Reviews and Meta-Analyses (PRISMA) flow diagram.**
**Supplemental Figure 2**	**Funnel plots of effect size versus standard error for studies evaluating the difference in PT, aPTT, D-Dimer, platelet count and fibrinogen.**
**Supplemental Table 1**	**Meta-regression analyses. Impact of age, male gender, hypertension, diabetes, and C-reactive protein (CRP) on the difference in PT, D-Dimer, platelet count, and fibrinogen levels.**
**Supplemental Table 3**	**PubMed search (June 16, 2020).**

Journal of
Clinical Medicine

MDPI

Supplemental Figure 1. Preferred Reporting Items for Systematic Reviews and Meta-Analyses (PRISMA) flow diagram.

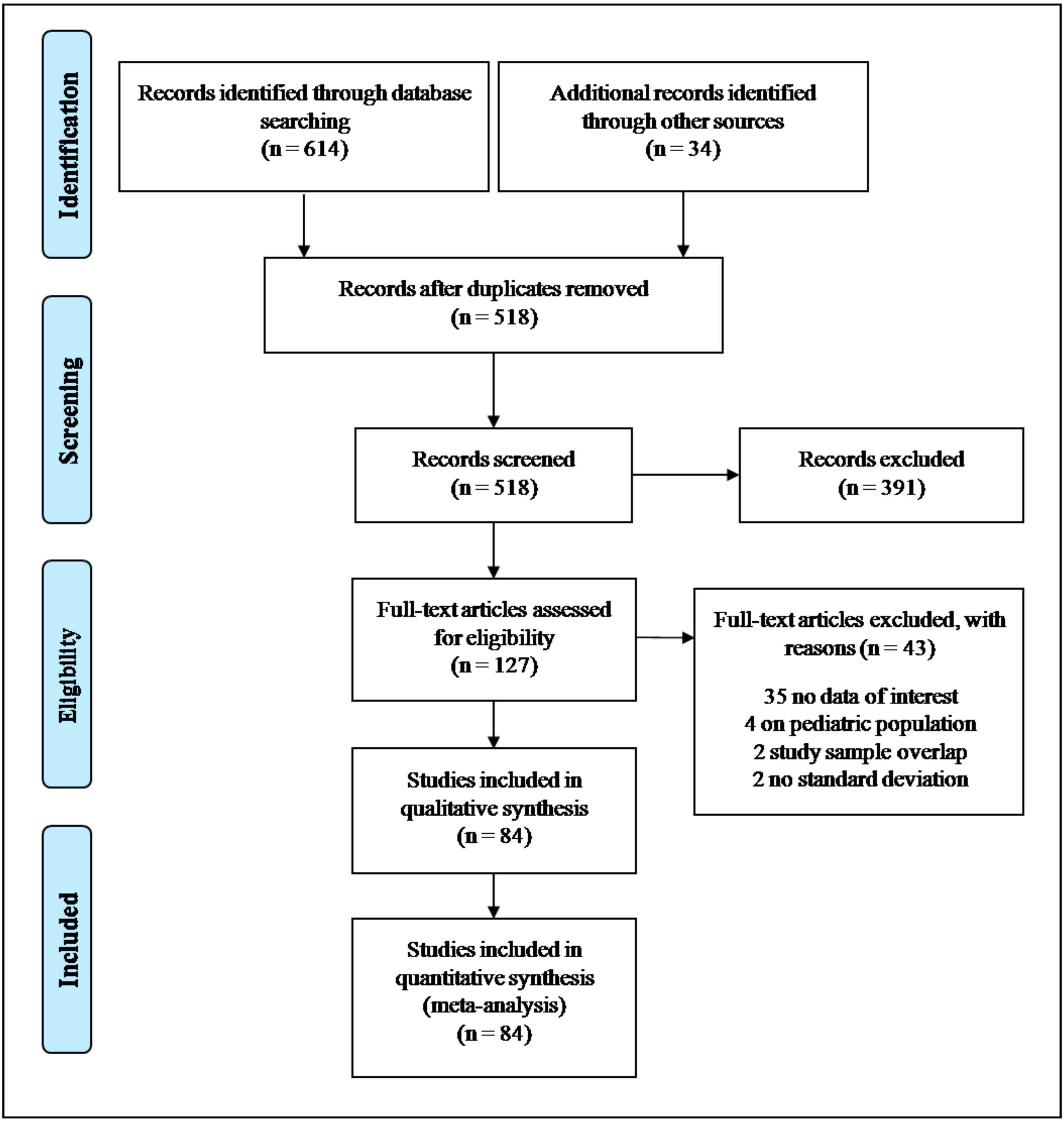

Journal of Clinical Medicine
MDPI

Supplemental Figure 2. Funnel plots of effect size versus standard error for studies evaluating the difference in prothrombin time (PT), activated partial thromboplastin time (aPTT), D-Dimer, platelet count and fibrinogen.

PT

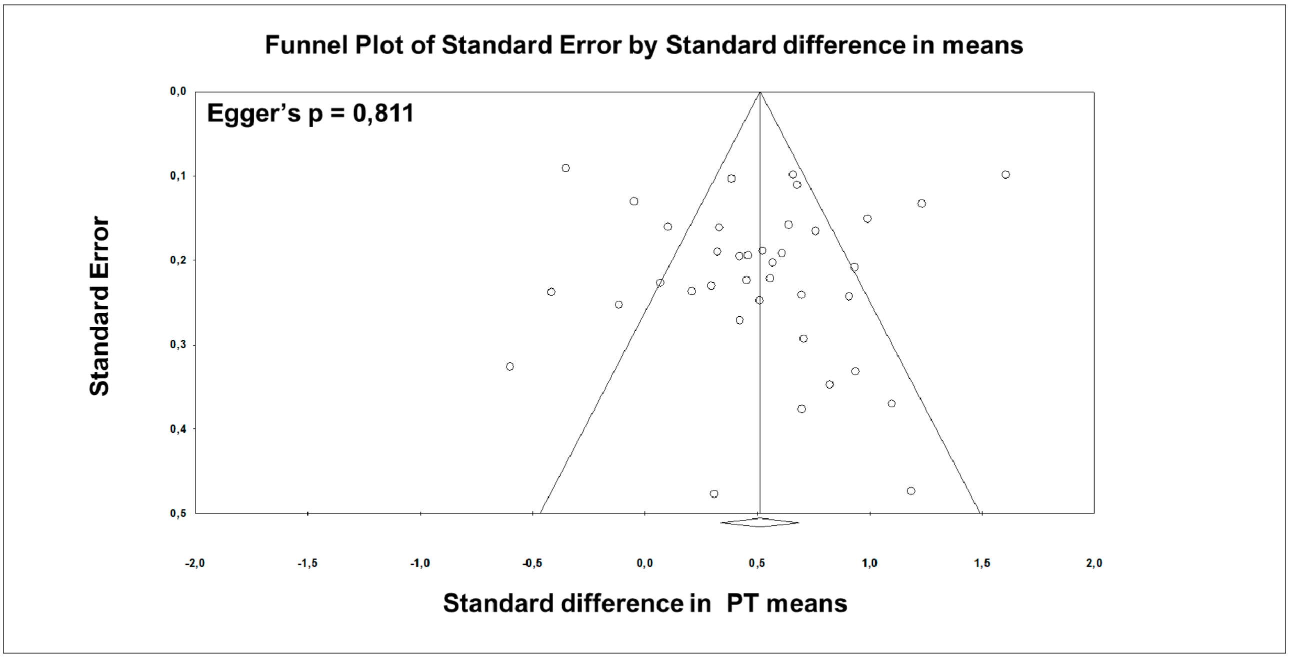

aPTT

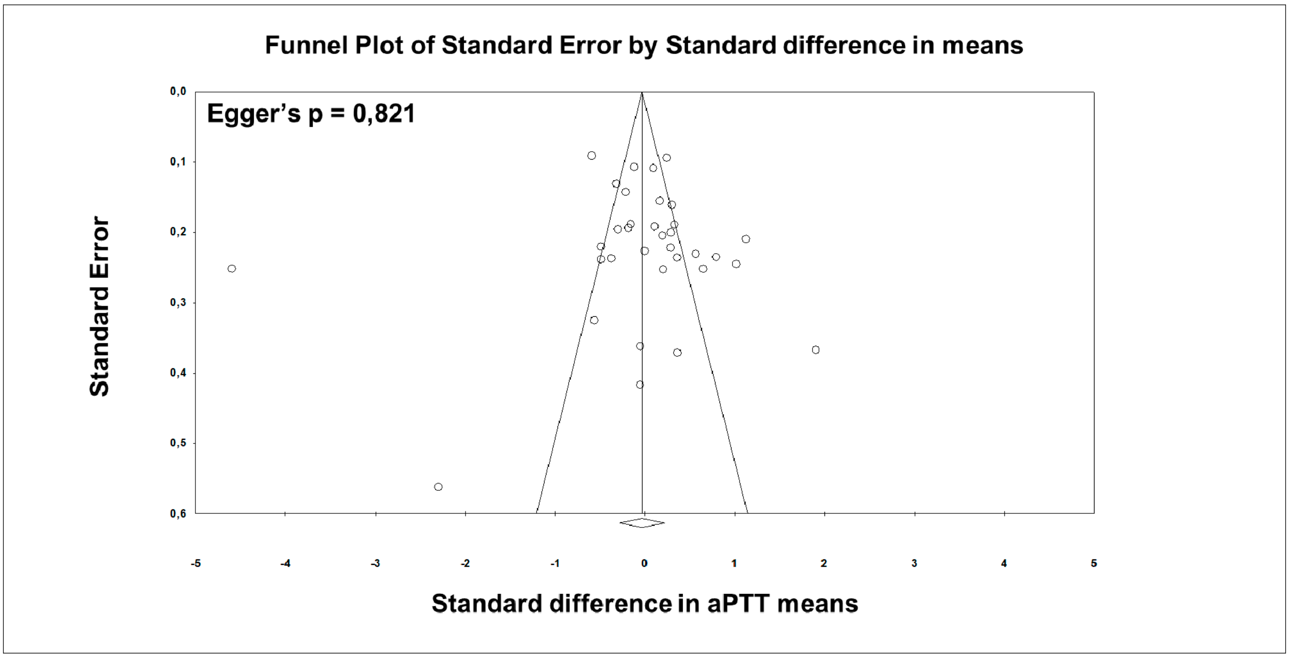

D-Dimer

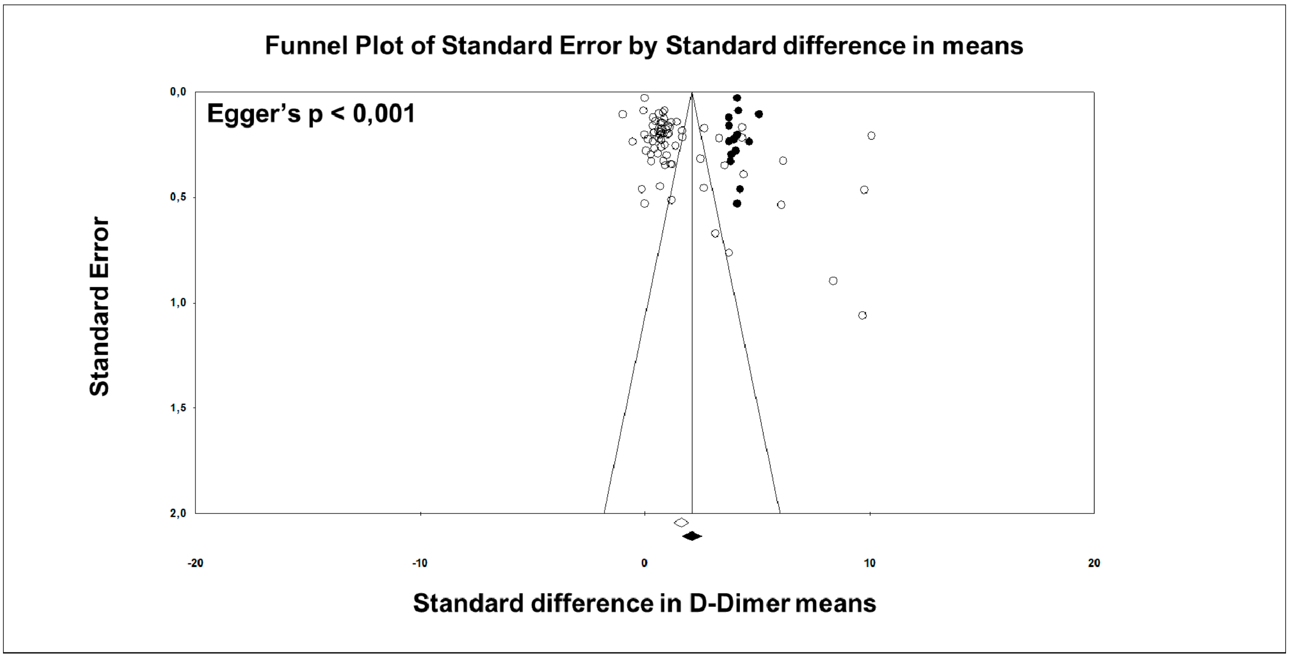

Platelet count

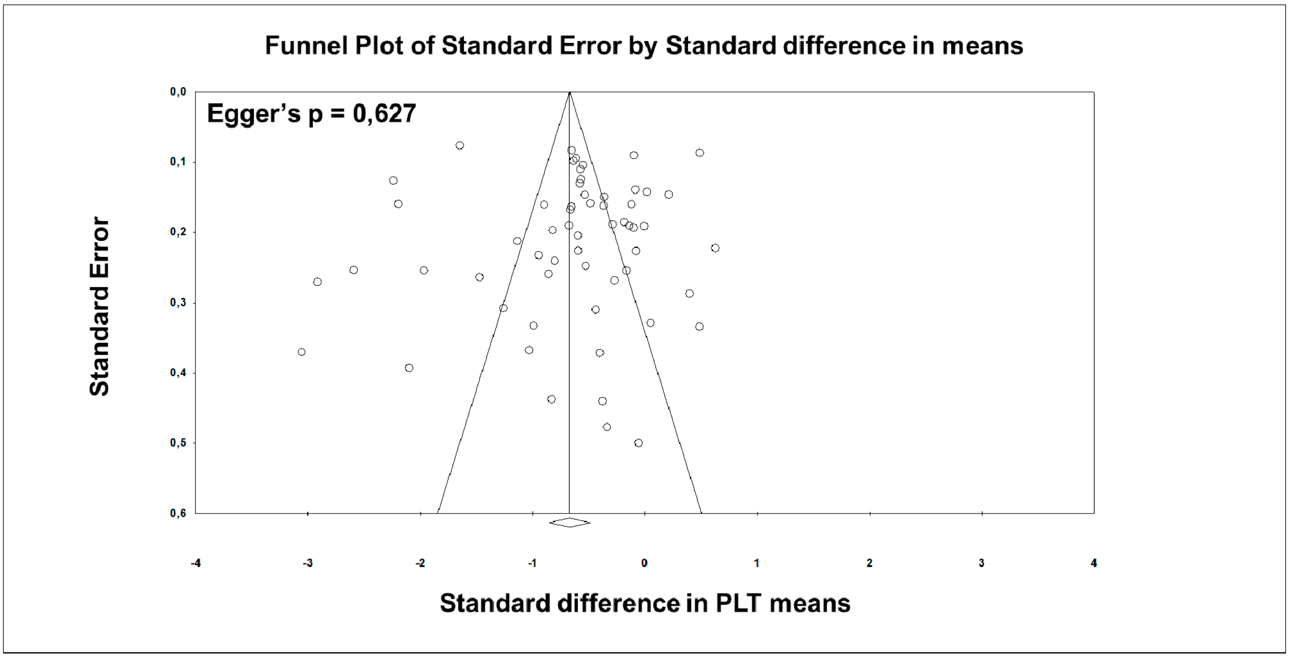

Journal of
Clinical Medicine

MDPI

Fibrinogen

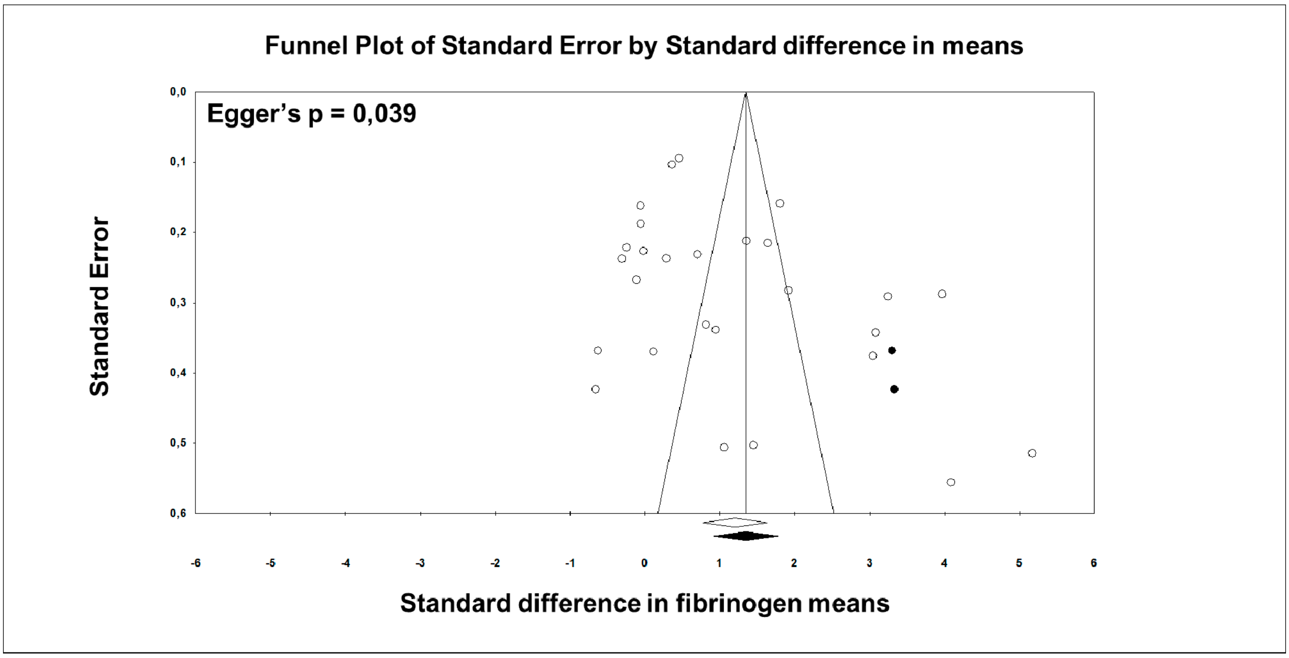

Journal of
Clinical Medicine

MDPI

Supplemental Table 1. Meta-regression analyses. Impact of age, male gender, hypertension, diabetes, and C-reactive protein (CRP) on the difference in PT, D-Dimer, platelet count, and fibrinogen levels.

Dependent variables		Independent variables				
		Age	Male sex	Hypertension	Diabetes	CRP
**PT**	**Z-value**<br>**P-value**	1.04<br>0.299	-0.11<br>0.915	-0.65<br>0.513	-0.08<br>0.935	**2.27**<br>**0.023**
**aPTT**	**Z-value**<br>**P-value**	-1.20<br>0.229	-1.28<br>0.202	1.03<br>0.301	-0.85<br>0.396	1.01<br>0.314
**D-Dimer**	**Z-value**<br>**P-value**	-0.81<br>0.418	**-2.43**<br>**0.015**	-0.22<br>0.829	1.43<br>0.154	-1.15<br>0.249
**PLT**	**Z-value**<br>**P-value**	0.53<br>0.595	**1.97**<br>**0.048**	-0.41<br>0.684	-0.76<br>0.450	0.50<br>0.619
**Fibrinogen**	**Z-value**<br>**P-value**	-0.77<br>0.440	0.18<br>0.854	**2.63**<br>**0.009**	-1.35<br>0.177	**2.89**<br>**0.004**

Legend: CRP: C-reactive protein; PT: prothrombin time; aPTT partial thromboplastin PLT: platelet count.

Journal of
Clinical Medicine

Supplemental Table 2. PubMed search (June 16, 2020).

**Keywords**	**Number of results**
(COVID-19 OR SARS-CoV-2)	22,792
(COVID-19 OR SARS-CoV-2) AND (coagulative)	226
(COVID-19 OR SARS-CoV-2) AND (coagulative OR coagulation)	226
(COVID-19 OR SARS-CoV-2) AND (coagulative OR coagulation OR haemostatic)	257
(COVID-19 OR SARS-CoV-2) AND (coagulative OR coagulation OR haemostatic OR hemostasis)	279
(COVID-19 OR SARS-CoV-2) AND (coagulative OR coagulation OR haemostatic OR hemostasis OR prothrombin time)	290
(COVID-19 OR SARS-CoV-2) AND (coagulative OR coagulation OR haemostatic OR hemostasis OR prothrombin time OR activated partial thromboplastin time)	292
(COVID-19 OR SARS-CoV-2) AND (coagulative OR coagulation OR haemostatic OR hemostasis OR prothrombin time OR activated partial thromboplastin time OR platelet)	369
(COVID-19 OR SARS-CoV-2) AND (coagulative OR coagulation OR haemostatic OR hemostasis OR prothrombin time OR activated partial thromboplastin time OR platelet OR D-Dimer)	477
(COVID-19 OR SARS-CoV-2) AND (coagulative OR coagulation OR haemostatic OR hemostasis OR prothrombin time OR activated partial thromboplastin time OR platelet OR D-Dimer OR fibrinogen)	484
